# Effect of cerebral sinus venous thrombosis and its location on cerebral blood flow: a [^15^O]water PET study in acute stroke patients compared to healthy volunteers

**DOI:** 10.1186/s13550-024-01180-9

**Published:** 2024-11-21

**Authors:** Andreas Harloff, Ganna Blazhenets, Johannes Fostitsch, Christoph Strecker, Rick Dersch, Ernst Mayerhofer, Philipp T. Meyer

**Affiliations:** 1 Department of Neurology and Neurophysiology, Medical Center, University of Freiburg, Faculty of Medicine, Breisacherstr. 64, 79106 Freiburg, Germany; 2 Department of Nuclear Medicine, Medical Center, University of Freiburg, Faculty of Medicine, Freiburg, Germany

**Keywords:** Cerebral sinus venous thrombosis, Cerebral blood flow, PET, [^15^O]water

## Abstract

**Background:**

Symptoms in acute cerebral sinus venous thrombosis (CSVT) are highly variable, ranging from headaches to fatal stroke, and the basis for this high inter-individual variability is poorly understood. The present study aimed to assess whether acute CSVT significantly alters regional cerebral blood flow (CBF), if findings differ from CBF patterns know from large-artery occlusion in stroke, and whether the pattern of CBF alterations depends on clot location. Therefore, we retrospectively analyzed 12 patients with acute CSVT 10.6 ± 4.6 days after symptom onset and ten healthy volunteers who underwent [^15^O]water PET (two scans each, 300 ± 14 MBq [^15^O]water). Static image datasets (15–75 s after injection; normalized to cerebellum) reflecting relative CBF (rCBF) were analyzed using voxel- and region-of-interest-based analysis (AAL3-atlas). We mirrored datasets of patients with left-sided CSVT to harmonize the affected hemisphere.

**Results:**

Seven and five patients showed right- and left-sided CSVT, respectively. The superior sagittal sinus (SSS) was involved in 8/12 patients. CSVT patients had extensive rCBF deficits in the voxel-based analysis with accentuation in the right (ipsilateral) frontal cortex and caudate nucleus compared to controls, which were most pronounced in cortical areas in those with involvement of the SSS (8/12), and in subcortical areas in those without involvement of the SSS (4/12; *p* < 0.05, false discovery rate corrected). ROI-analysis demonstrated significant frontal (*p* = 0.01) and caudate nucleus (*p* = 0.008) rCBF deficits driven by patients with and without SSS occlusion, respectively.

**Conclusions:**

[^15^O]water PET was able to visualize characteristic patterns of impaired rCBF, which were different from intracranial large-artery occlusion in acute ischemic stroke, and exhibited substantial rCBF alterations depending on the involvement of the SSS. Our findings provide novel insights into the effects of disturbed venous drainage on CBF in acute CSVT, which may aid in understanding the pathophysiology, and guide future therapy of acute CSVT.

## Background

Cerebral sinus venous thrombosis (CSVT) is a rare cause of stroke with an incidence of 1–2 per 100,000 person-years [1]. Symptoms in CSVT are highly variable ranging from headaches to fatal stroke. Complications include brain ischemia, hemorrhage, and even herniation in severe cases leading to a mortality of up to 8% [2]. It is tempting to speculate that this high inter-individual variability is related to systemic (e.g., age, comorbidities) and local factors like extent and location of clot formation and available venous collaterals (among others), which, however, are poorly understood [3, 4]. Common pathophysiology of acute ischemic stroke is brain ischemia downstream to the site of an artery occlusion. In contrast, CSVT causes disturbed drainage of venous blood and a fatal cascade of venous congestion with dilatation of the venous and capillary bed, reduced capillary perfusion pressure, edema, infarction, and intracranial bleeding upstream to the site of venous occlusion [5, 6].

Very few case studies that measured cerebral blood flow (CBF) in CSVT using computer tomography (CT) perfusion imaging and arterial spin labeling magnetic resonance imaging (MRI) found that CBF decreases in affected brain territories (for a recent review see [6]), which significantly improved on follow-up [7]. Furthermore, disrupted venous macro-circulation in CSVT was recently visualized using four-dimensional flow magnetic resonance imaging. This study visualized individual collateral veins and demonstrated the restoration of blood flow in cerebral sinuses following six months of oral anticoagulation [8]. Based on the current evidence [6, 7], the extent and magnitude of CBF deficits may be a valuable predictor of clinical outcome. However, positron emission tomography (PET) with the freely diffusible tracer [^15^O]water, as the reference method for assessment of regional CBF [9], has not yet been used to systematically investigate CBF changes in CSVT but has only been applied in single cases (e.g [10]).

Against this background, we undertook a retrospective study in patients with acute CSVT who underwent [^15^O]water PET to explore possible regional CBF alterations and their dependence on the location of the venous thrombus. We hypothesized, that regional CBF is significantly impaired in acute CSVT despite a broad network of intracranial venous collaterals if large sinuses are affected. Moreover, that patterns of regional CBF abnormalities in patients with CSVT differ from that in stroke patients with intracranial arterial occlusion (i.e., venous drainage territories vs. arterial supply territories), and that pattern of disturbed regional CBF is dependent on clot location in acute CSVT.

## Methods

Between October 2021 and June 2023, 24 consecutive patients with acute CSVT were admitted to our institution. Thirteen of these patients, who underwent [^15^O]water PET for examination of individual regional CBF, were eligible for the present retrospective analysis. However, one patient was previously treated for a frontal glioblastoma. Because of residual changes in the frontal lobe, this patient was excluded from further analysis, leaving a total of twelve patients being included. [^15^O]water PET was not performed in the other eleven patients for the following reasons: five were in a bad clinical condition requiring prolonged intensive care or had a severe prognosis due to associated metastatic cancer. Three patients refused to undergo [^15^O]water PET as an additional imaging procedure, one patient was pregnant and one patient had to be isolated due to acute SARS-CoV2 infection. Ten healthy controls (HC) from an ongoing prospective study scanned under comparable conditions served as a control group. Scans were acquired in duplicate to account for the relatively high noise level of [^15^O]water PET.

After diagnosis of CSVT by contrast-enhanced MRI or CT venography, all patients received immediate therapeutic anticoagulation with unfractionated intravenous or low molecular weight heparin when admitted to our stroke unit or neurological intensive care unit. Parenteral anticoagulation was replaced by oral anticoagulation with vitamin K antagonists (INR 2.0–3.0) or off-label treatment with dabigatran (2 × 110/150 mg per day) at discharge from hospital and continued for at least six months. Follow-up brain MRI and neurological examination were performed six months later in 6/12 patients. The study has been approved by the institutional review board and the need for written informed consent to this retrospective analysis was waived (vote no. 23-1549-S1-retro).

### PET imaging

Two 4-min list mode scans under resting conditions at ambient light and noise were acquired in patients and healthy controls, starting with intravenous injection of 300 ± 14 MBq [^15^O]water on a fully-digital PET/CT (Philips, Vereos). PET datasets were reconstructed employing low-dose CT for attenuation correction and the vendor-specific, line-of-response time-of-flight ordered-subsets 3-dimensional iterative reconstruction algorithm using spherically symmetric basis functions (so-called blob ordered-subset time-of-flight reconstruction; number of iterations, 5; number of subsets, 11; 2 mm Gaussian post filtering; point spread function off; resulting voxel size, 1.0 mm^3^), yielding an isotropic voxel size of 1 × 1 × 1 mm^3^. Static images over one minute after tracer arrival in the brain (i.e., 15–75 s) were calculated and normalized to the cerebellum to gain maps of relative CBF (rCBF). These maps were used for further voxel- and region-of-interest (ROI)-based analyses (see below). We mirrored image datasets of patients with left-sided CSVT to harmonize the affected hemisphere (i.e., right).

### Image analysis

All preprocessing steps were implemented with an in-house pipeline in MATLAB (The MathWorks, Inc.) and Statistical Parametric Mapping (SPM) 12 software (www.fil.ion.ac.uk/spm). For each individual, the second PET scan was realigned to the first PET scan to correct for possible inter-scan misalignment. Realigned scans were spatially normalized to an in-house PET template in MNI space.

Voxel-wise comparisons between groups were performed in SPM using full-factorial design accounting for repeated measures. For this analysis, rCBF maps were smoothed with an isotropic Gaussian kernel of 10 mm full-width at half maximum. Voxels with false-discovery rate (FDR)-corrected *p* < 0.05 and a cluster extent of at least 0.1 mL (corresponding to 100 voxels) were considered statistically significant.

**Fig. 1:**
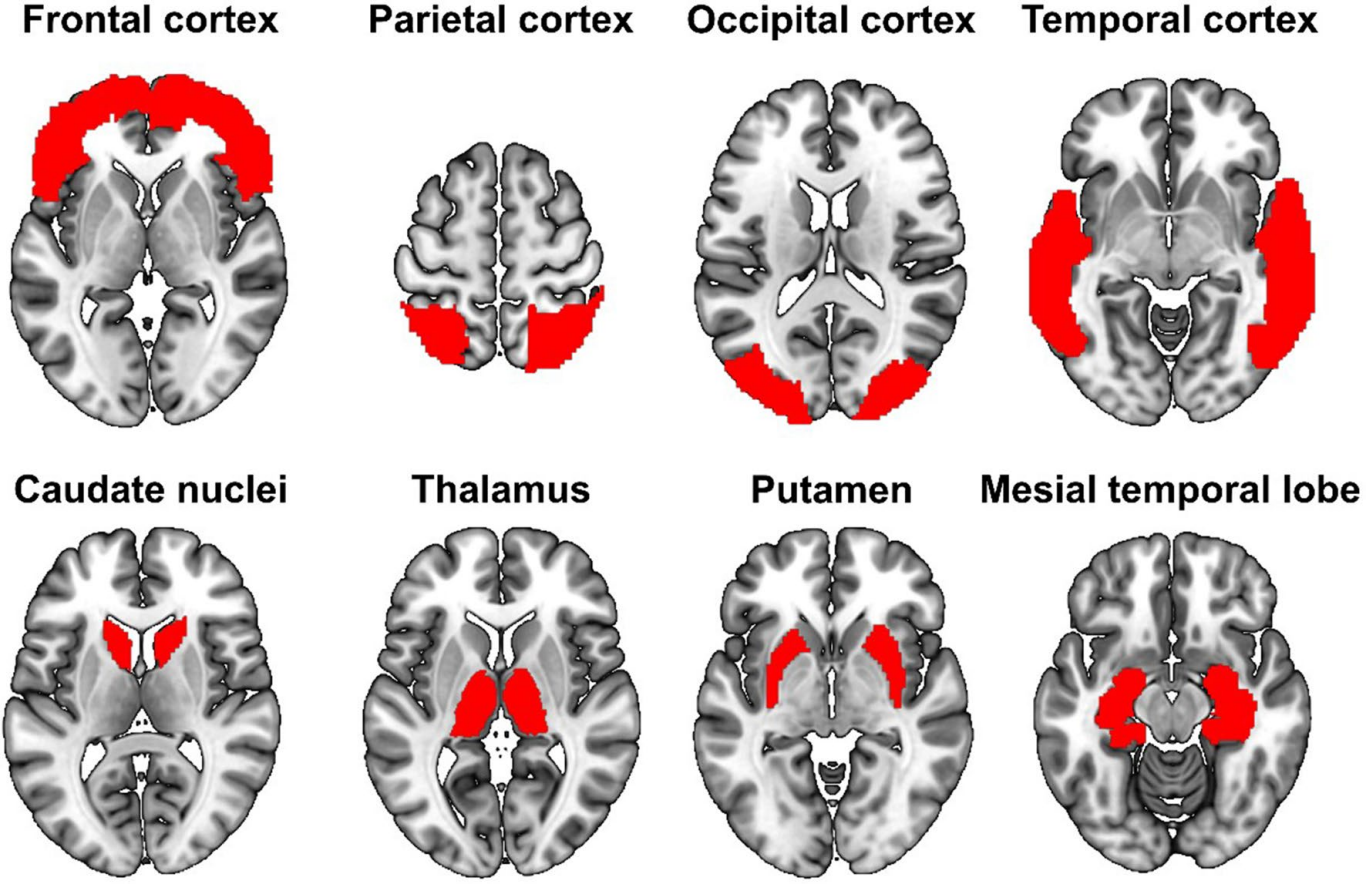
Anatomical definitions of the corresponding masks employed for region of interest analysis

Mean rCBF in regions from the automatic anatomic labeling (AAL3) atlas [11] was calculated in each patient and healthy control. Selected AAL3-defined regions were grouped to create bilateral cortical ROI as follows: frontal cortex (superior, middle, and inferior frontal gyri), parietal cortex (superior and inferior parietal gyri), occipital cortex (superior, middle, and inferior occipital gyri), temporal cortex (superior, middle, and inferior temporal gyri), and mesial temporal lobe (hippocampus, parahippocampal gyrus and amygdala). In addition, we assessed mean rCBF in bilateral caudate nucleus, putamen, and thalamus (see Fig. 1). Since spatial normalization and ROI placement on caudate nuclei may be inaccurate in patients with enlarged ventricles, we carefully checked the position of the caudate nucleus ROI and adjusted their position in mediolateral (x) direction if felt necessary.

### Statistical analysis

Statistical analyses were performed with R software (version 4.1.0, http://www.R-project.org/). Group differences in mean rCBF of aforementioned ROI were tested with repeated-measures ANOVA followed by pairwise least square means comparison with Tukey multiplicity adjustment. For all comparisons, adjusted *p* < 0.05 was considered statistically significant.

## Results

### Study cohort and MRI findings

Patients’ characteristics are given in Table 1. Seven and five patients showed right- and left-sided CSVT, respectively. The superior sagittal sinus (SSS) was involved in most patients (8/12, 66.7%). On cerebral MRI, two patients showed venous congestion with edema, one patient showed intracerebral hemorrhage, and one showed bilateral thalamic infarction. Patients (54.1 ± 17.5 years) were significantly older than healthy controls (29.5 ± 9.0 years; *p* = 0.001). [^15^O]water PET was performed 11 ± 5 days after the onset of CSVT symptoms (Table 1).

**Table 1:** Characteristics of patients with cerebral sinus venous thrombosis and healthy controls

Characteristics	Value
Age of patients – mean (SD)	54.1 (17.5) years
Female sex of patients – n (%)	6 (50.0)
Time between symptom onset and PET – mean (SD)	10.6 (4.6) days
Site of venous occlusions* – n (%)	
Superior sagittal sinus	8 (66.7)
Right transverse sinus	7 (58.3)
Left transverse sinus	5 (41.7)
Internal cerebral veins	1 (8.3)
Right-sided cortical veins	2 (16.7)
Left-sided cortical veins	1 (8.3)
Age of heathy volunteers – mean (SD)	29.5 (9.0) years
Female sex of healthy controls – n (%)	6 (60.0)

*Most patients had venous occlusions at multiple sites. SD = standard deviation

### Results of regional cerebral blood flow (rCBF) analysis

**Fig. 2:**
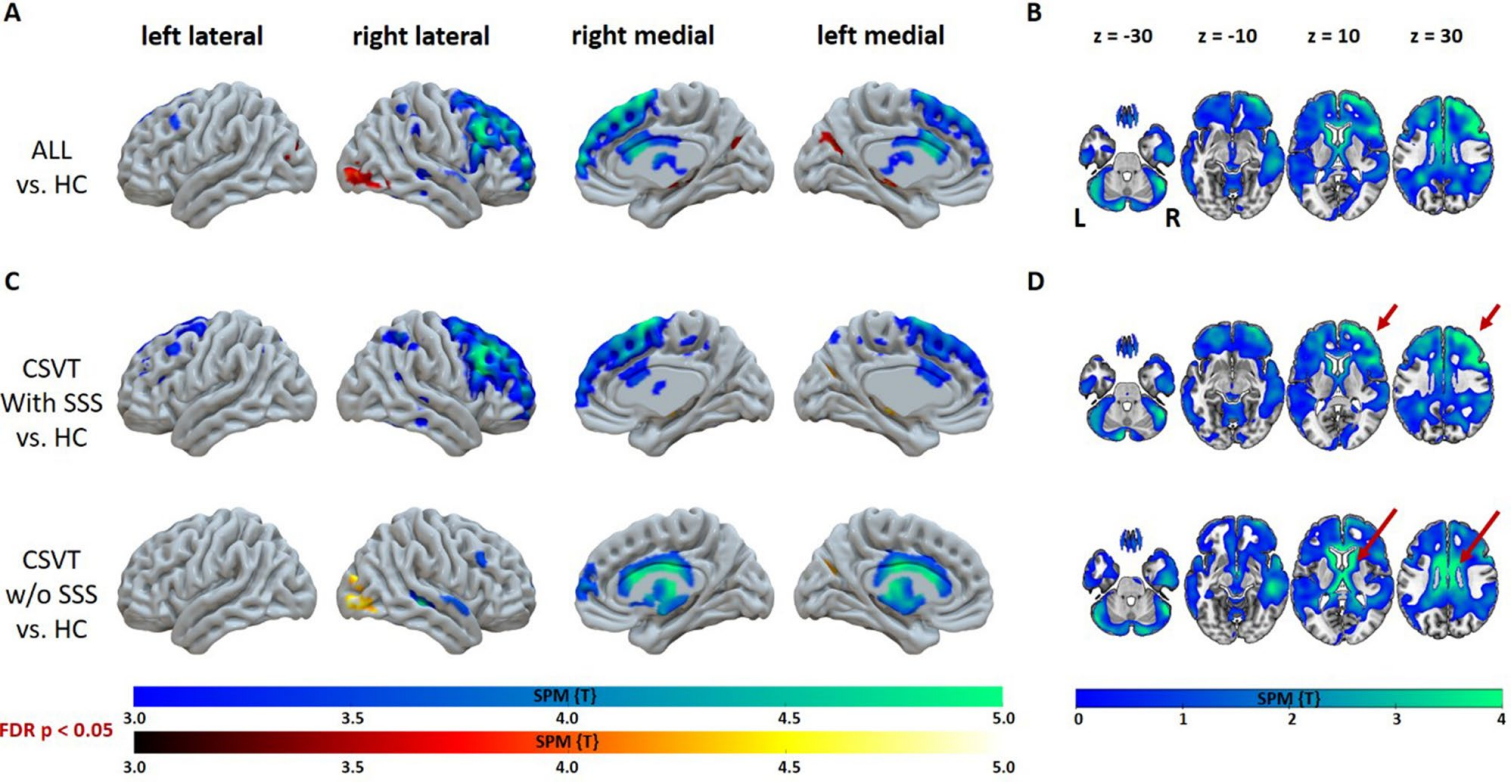
Regional changes in relative cerebral blood flow in patients with cerebral sinus venous thrombosis (CSVT) compared to healthy controls (HC). Surface overlays of T-maps thresholded at *p* < 0.05, false discovery rate (FDR)-corrected (**A**, **C**); cold and hot colors correspond to decreased and increased rCBF, respectively. To illustrate the extent of rCBF decrease, selected axial slices depict non-thresholded negative T-maps (**B**, **D**). Results are shown for the whole group (**A**, **B**) and the sub-cohorts of patients with and without (w/o) superior sagittal sinus (SSS) occlusion (**C**, **D**). Arrows point to dominant cortical and subcortical findings. SPM = Statistical Parametric Mapping

Voxel-wise comparison to healthy controls indicated extensive rCBF deficits in CSVT patients being most accentuated in the frontal cortex and caudate nucleus (*p* < 0.05, false discovery rate (FDR)-corrected, Fig. 2). The cortical finding was lateralized to the ipsilateral side (more affected hemisphere harmonized to the right). When split based on the site of venous occlusion, patients with involvement of SSS showed pronounced cortical deficits, while those without SSS thrombosis showed a stronger subcortical pattern (see Fig. 2C and D). The reverse contrast (higher rCBF in CSVT patients compared to healthy controls) revealed right-dominant occipital clusters of increased rCBF (*p* < 0.05, FDR-corrected) that were driven by the patients without SSS thrombosis (Figs. 1 and 2).

**Fig. 3:**
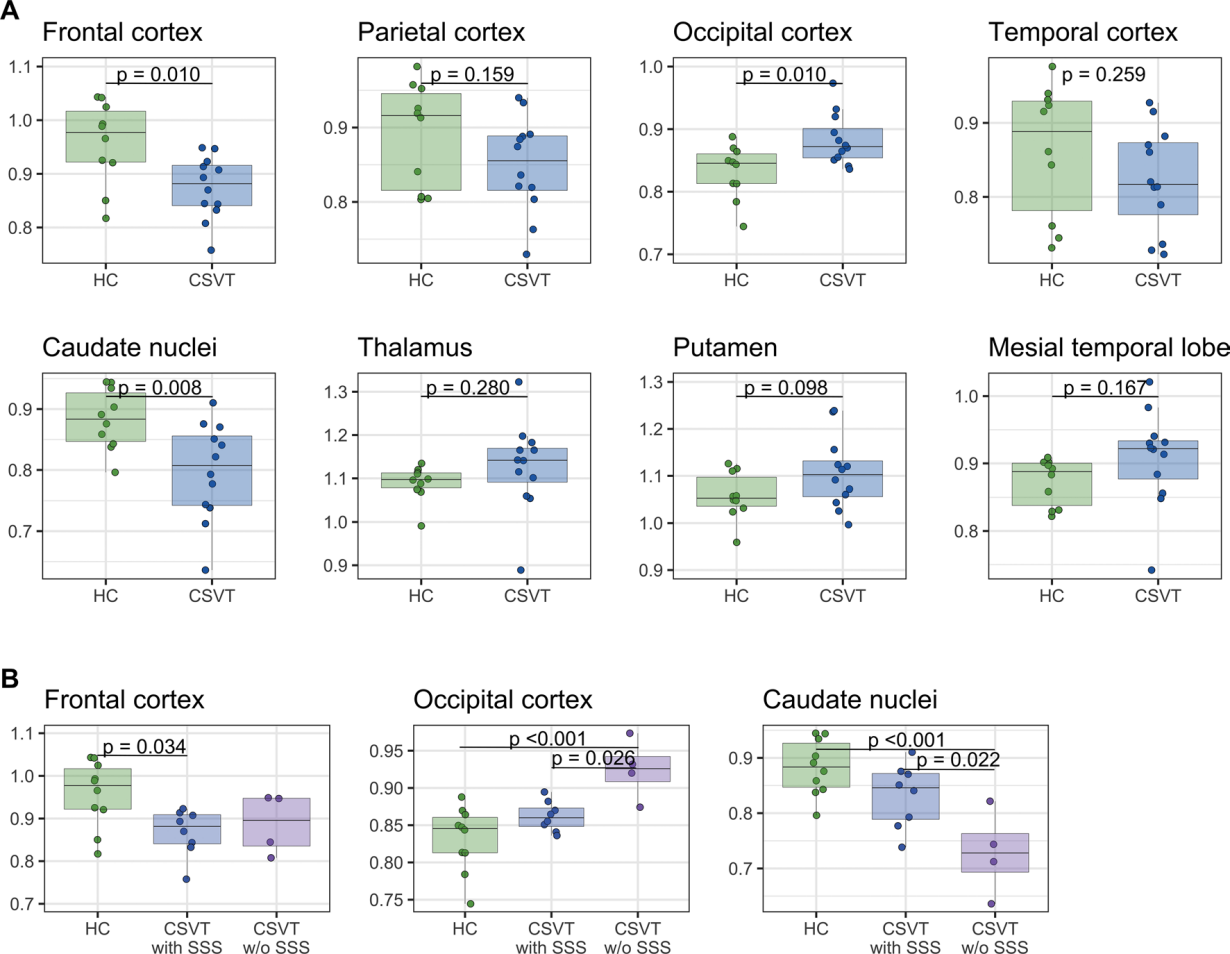
Region of interest analysis of relative cerebral blood flow (rCBF). **A**: rCBF in all patients with cerebral sinus venous thrombosis (CSVT) compared to healthy controls (HC). **B**: rCBF in subgroups of patients with cerebral sinus venous thrombosis with and without (w/o) thrombosis of the superior sagittal sinus (SSS). Values are averaged by repetition

ROI analysis demonstrated significant frontal (F_1,20_ = 8.11, *p* = 0.010) and caudate nucleus (F_1,20_ = 8.62, *p* = 0.008) rCBF deficits in CSVT patients (Fig. 3). Considering the subgroups, deficits in frontal cortex were significant only in patients with thrombosis of the SSS (t_19_ = -2.71, *p* = 0.034, relative difference between SVT patients with SSS thrombosis and healthy controls = -9.3%). In turn, deficits in caudate nucleus were only significant in those without thrombosis of the SSS (t_19_ = -4.51, *p* < 0.001, relative difference between SVT patients without SSS thrombosis and healthy controls = -17.4%). The latter group also showed an occipital rCBF increase (t_19_ = 4.36, *p* < 0.001, relative difference between SVT patients without SSS thrombosis and healthy controls = 11.2%). Other regions showed no significant differences. No significant effect of repetition was found for any model (all *p* > 0.05). In an exploratory analysis, regional rCBF values of the ipsilateral and contralateral hemispheres were assessed by separate repeated-measures ANOVA models. In analogy to bilateral ROI analyses, between-group differences were significant for both ipsi- and contralateral hemispheres in frontal (F = 8.52, *p* = 0.008 and F = 6.76, *p* = 0.017, respectively) and caudate nuclei (F = 7.10, *p* = 0.015 and F = 9.07, *p* = 0.007, respectively), while differences in the occipital lobe were only significant on the ipsilateral side (F = 10.12, *p* = 0.005).

### Neurological follow-up

At six months follow-up, four of six evaluated patients were free of neurological deficits and headaches. One patient (normal cerebral MRI, left hemispheric focus on electroencephalogram) reported a slight impairment of memory and fine motor skills of her right hand. Another patient with temporooccipital intracerebral hemorrhage reported persistent mild amnesic aphasia and paraphasia.

## Discussion

We report the first systematic study on CBF changes as assessed by [^15^O]water PET in patients with acute CSVT. Our findings provide new insights into the pathophysiology of this rare but serious disease. Despite the limited number of patients with acute CSVT, we identified robust patterns of disturbed CBF by voxel- and ROI-based analyses.

The decrease of rCBF predominantly in frontal lobes in eight patients with SSS thrombosis is plausible: SSS occlusion hampers drainage of superficial cortical veins leading to venous congestion and consecutive CBF decrease in the upstream brain tissue (frontal and possibly parietal lobes) [5]. CBF patterns in the four patients with open SSS but thrombosed lateral sinuses and/or internal cerebral veins were different: CBF decreased predominately in the basal ganglia, thalami, internal capsules, and mesiotemporal lobes. These patterns fit venous drainage territories described in the literature [6] and are strikingly different from the patterns on [^15^O]water PET in patients with intracranial arterial occlusion, the etiology of the vast majority of strokes [12]. Moreover, on voxel-wise analyses cortical findings were lateralized to the ipsilateral side (harmonized right-sided CSVT). In regional analyses, we observed significant differences bilaterally, with no laterality effects (except occipital cortex in exploratory analysis), which we attribute to the relatively large cortical ROIs used. Interestingly, we also found a CBF increase in the occipital lobe in patients without SSS thrombosis. Even though not yet been reported in humans before, Nakase et al. [13] found hyperperfused zones around hypoperfused cores in a rat model of CSVT up to 30 min after cortical vein occlusion. Our patients were scanned at a considerably later stage (days after occlusion). Thus, the basis of this observation remains obscure, and several possible explanations remain (e.g., collateralization through capillary bed, hyperperfusion upon reperfusion, artifact), warranting future studies.

Limitations of the present study are the small cohort (also limiting in-depth subgroup analyses and symptom-imaging correlations), and the younger age of the healthy controls. Due to its low incidence, recruiting patients with acute CSVT is challenging even in a tertiary center. Larger cohorts need to be recruited by multi-center studies with access to [^15^O]water PET. Such studies should also include detailed neuropsychological assessments and standardized follow-ups. This would allow one to gain further insights into the pathophysiology, detect (possibly subclinical) cognitive impairments, and test the prognostic value of [^15^O]water PET in acute CSVT (as expected in analogy to perfusion MRI and CT [6, 7]). While early PET studies using low-resolution scanners provided conflicting data on CBF decrease with age [reviewed in 7], a study using a moderate-resolution scanner demonstrated that a weak negative correlation between cortical (not subcortical) CBF and age is due to atrophy-related partial volume effects [14]. It is unlikely that the younger age of healthy controls explains the rCBF deficits in CSVT in the present study as we found marked and well-differentiated patterns of rCBF changes (decreases and increases) according to side (more pronounced ipsilaterally) and location (cortical vs. subcortical) of CSVT. Furthermore, we used a state-of-the-art fully digital PET/CT scanner providing superior spatial resolution (allowing for quantitative imaging of small brain structures [15]) and, thus, should be much less affected by partial volume effects. Finally, regional measures were normalized to the cerebellum to gain maps of rCBF. Thus, CSVT-associated alterations of cerebellar CBF may have contributed to observed rCBF changes. However, we consider this highly unlikely because cerebellar lesions or symptoms were found in only 5% of patients with CSVT in a previous study [16]. Consistently, none of our 12 CSVT patients had cerebellar infarction or hemorrhage on brain MRI. In addition, a bias induced by rCBF scaling should lead to homogenous global and not regional changes.

## Conclusions

The present study demonstrates that patients with acute cerebral sinus venous thrombosis exhibit substantial rCBF alterations, which are strikingly different from rCBF patterns in intracranial artery occlusion and dependent on the location of cerebral sinus venous thrombosis, in particular on the involvement of the superior sagittal sinus. These findings may aid in understanding the pathophysiology and guide future therapy of acute cerebral sinus venous thrombosis. Future prospective studies in larger cohorts of patients undergoing standardized neurological follow-up are needed in order to evaluate the potential prognostic value of [^15^O]water PET in this important disease.

## Data Availability

The datasets used and/or analyzed during the current study are available from the corresponding author on reasonable request.

## Abbreviations

AAL3: Automatic anatomic labeling
ANOVA: Analysis of variance
CBF: Cerebral blood flow
rCBF: Regional cerebral blood flow
CT: Computer tomography
FDR: False-discovery rate
INR: International normalized ratio
MATLAB: MATrix LABoratory
MNI: Montreal Neurological Institute
MRI: Magnetic resonance imaging
PET: Positron emission tomography
ROI: Region of interest
SPM: Statistical parametric mapping
SSS: Superior sagittal sinus
